# Genetic evidence for a causal relationship between type 2 diabetes and peripheral artery disease in both Europeans and East Asians

**DOI:** 10.1186/s12916-022-02476-0

**Published:** 2022-08-31

**Authors:** Xuehao Xiu, Haoyang Zhang, Angli Xue, David N. Cooper, Li Yan, Yuedong Yang, Yuanhao Yang, Huiying Zhao

**Affiliations:** 1 Department of Medical Research Center, Sun Yat-sen Memorial Hospital, Sun Yat-sen University; Guangdong Provincial Key Laboratory of Malignant Tumor Epigenetics and Gene Regulation, Guangzhou, China; 2 School of Data and Computer Science, Sun Yat-sen University, Guangzhou, 510000 China; 3 Garvan-Weizmann Centre for Cellular Genomics, Garvan Institute of Medical Research, Sydney, NSW Australia; 4 Institute for Molecular Bioscience, The University of Queensland, Brisbane, QLD Australia; 5 Institute of Medical Genetics, School of Medicine, Cardiff University, Heath Park, Cardiff, CF14 4XN UK; 6 Mater Research Institute, Translational Research Institute, Brisbane, QLD Australia

**Keywords:** Type 2 diabetes, Peripheral artery disease, Shared genetics, Causality

## Abstract

**Background:**

Observational studies have revealed that type 2 diabetes (T2D) is associated with an increased risk of peripheral artery disease (PAD). However, whether the two diseases share a genetic basis and whether the relationship is causal remain unclear. It is also unclear as to whether these relationships differ between ethnic groups.

**Methods:**

By leveraging large-scale genome-wide association study (GWAS) summary statistics of T2D (European-based: *N*_case_ = 21,926, *N*_control_ = 342,747; East Asian-based: *N*_case_ = 36,614, *N*_control_ = 155,150) and PAD (European-based: *N*_case_ = 5673, *N*_control_ = 359,551; East Asian-based: *N*_case_ = 3593, *N*_control_ = 208,860), we explored the genetic correlation and putative causal relationship between T2D and PAD in both Europeans and East Asians using linkage disequilibrium score regression and seven Mendelian randomization (MR) models. We also performed multi-trait analysis of GWAS and two gene-based analyses to reveal candidate variants and risk genes involved in the shared genetic basis between T2D and PAD.

**Results:**

We observed a strong genetic correlation (*r*_*g*_) between T2D and PAD in both Europeans (*r*_g_ = 0.51; *p*-value = 9.34 × 10^−15^) and East Asians (*r*_*g*_ = 0.46; *p*-value = 1.67 × 10^−12^). The MR analyses provided consistent evidence for a causal effect of T2D on PAD in both ethnicities (odds ratio [OR] = 1.05 to 1.28 for Europeans and 1.15 to 1.27 for East Asians) but not PAD on T2D. This putative causal effect was not influenced by total cholesterol, body mass index, systolic blood pressure, or smoking initiation according to multivariable MR analysis, and the genetic overlap between T2D and PAD was further explored employing an independent European sample through polygenic risk score regression. Multi-trait analysis of GWAS revealed two novel European-specific single nucleotide polymorphisms (rs927742 and rs1734409) associated with the shared genetic basis of T2D and PAD. Gene-based analyses consistently identified one gene *ANKFY1* and gene-gene interactions (e.g., *STARD10* [European-specific] to *AP3S2* [East Asian-specific]; *KCNJ11* [European-specific] to *KCNQ1* [East Asian-specific]) associated with the trans-ethnic genetic overlap between T2D and PAD, reflecting a common genetic basis for the co-occurrence of T2D and PAD in both Europeans and East Asians.

**Conclusions:**

Our study provides the first evidence for a genetically causal effect of T2D on PAD in both Europeans and East Asians. Several candidate variants and risk genes were identified as being associated with this genetic overlap. Our findings emphasize the importance of monitoring PAD status in T2D patients and suggest new genetic biomarkers for screening PAD risk among patients with T2D.

**Supplementary Information:**

The online version contains supplementary material available at 10.1186/s12916-022-02476-0.

## Introduction

Type 2 diabetes (T2D) is a severe metabolic disease caused primarily by the inadequate production or secretion of insulin [1]. Previous studies have revealed that T2D patients have a significantly increased risk of suffering from peripheral artery disease (PAD) [2, 3], a chronic circulatory condition characterized by the constriction of arteries that serves to slow the blood flow in the limbs [4, 5]. Evidence has accumulated for a higher mortality rate among patients with co-morbid T2D and PAD than among patients with PAD alone [6], illustrating the importance of understanding the pathophysiological link between T2D and PAD.

The observed association between T2D and PAD can be explained in part by a shared genetic basis [7–9]. For example, Strawbridge and van Zuydam identified *CDKN2A/B* as candidate genes likely to be involved in regulating both T2D and PAD [8]. A recent study also replicated *CDKN2A* as being associated with PAD in individuals with diabetes [10]. Vujkovic et al. analyzed ~ 1.4 million individuals of multiple ethnicities (including Europeans, African Americans, Hispanics, South Asians, and East Asians), thereby disclosing significant associations between 318 genetic variants and the risk of both T2D and PAD [9]. Nevertheless, our knowledge of the nature and extent of the genetic associations between T2D and PAD is still far from clear. Furthermore, whether the associations between T2D and PAD differ among ethnic groups remains uncertain, and whether the genetic associations between the two diseases reflect a causal relationship or pleiotropy is unknown.

The growth of genome-wide association studies (GWAS) over the past two decades has stimulated the development of approaches to analyze cross-trait shared genetic architecture based on GWAS summary statistics. For example, Bulik-Sullivan et al. proposed the GWAS-based linkage disequilibrium score regression (LDSC) statistic as a means to estimate the single nucleotide polymorphism (SNP)-based genetic correlation between traits [11]. Mendelian randomization (MR) has been proposed as a way to measure the putative causal relationship between traits. MR uses genetic variants as instruments to mimic a random allocation procedure in randomized controlled trials [12], thereby avoiding issues of confounding and reverse causation [13]. With the advent of large-scale biobanks involving multiple ethnicities (e.g., UK Biobank [UKB], BioBank Japan [BBJ]), it became possible to compare the cross-trait shared genetic architecture between different ethnicities in order to explore the possibility of trans-ethnic genetic heterogeneity. To date, the applications of these approaches and datasets have achieved considerable success in increasing our understanding of the shared genetic architecture between complex diseases [14–16], thereby superseding traditional experimental models which are much more time-consuming and costlier to implement. Thus, using GWAS-based approaches to analyze GWAS summary data for T2D and PAD derived from different ethnic groups provides an opportunity for us to study the shared genetic basis between the two diseases.

In this study, we applied LDSC and seven MR or MR-equivalent approaches to estimate the genetic correlation and potential causal relationship between T2D and PAD in Europeans and East Asians. Further multivariable Mendelian randomization analysis (MVMR) was used to examine whether the putative causal relationship between T2D and PAD can be affected by traits associated with the increased risk of PAD or T2D. A validation of genetic overlap between T2D and PAD was then performed using independent samples to explore whether the polygenic risk scores (PRS) for T2D could predict the status of PAD, and vice versa. Finally, we performed a multi-trait analysis of GWAS (MTAG) and two gene-based analyses (i.e., multi-marker analysis of genomic annotation [MAGMA] and summary data-based Mendelian randomization [SMR]) to identify the risk SNPs and functional genes that are likely to be responsible for the shared genetic etiology underlying T2D and PAD. We applied the analytical pipeline to Europeans, and also to East Asians independently as a replicated analysis, to investigate the common and distinct mechanisms of co-occurring T2D-PAD between Europeans and East Asians.

## Methods

### Data sources for Europeans

Analyses were performed by utilizing more than 400,000 individuals of European ancestry from the UKB cohort. Individuals of European ancestry were selected using a two-stage approach: first, we calculated the top two principal components (PCs) for each individual from HapMap 3 (HM3) SNPs of 1000 Genomes Project [17]; second, we assigned individuals to European if their PC information was optimally matched to the 1000 European Genomes reference (compared to other population reference, e.g., African, East Asian, South Asian and Admixed) [18]. Individuals were classified as T2D or PAD patients if they were coded with the International Classification of Diseases 10th (ICD-10) based on non-insulin-dependent (type 2) diabetes mellitus (UKB Field ID: 41270 [E11]; Additional file 1: Table S1) or ICD-10 based diseases of arteries, arterioles, and capillaries (UKB Field ID: 41270 [I70-I79]; Additional file 1: Table S1), respectively [19]. They were classified as controls in relation to T2D or PAD if they had not been ascribed the corresponding T2D or PAD code, respectively. In total, we obtained a T2D sample of 364,673 individuals (*N*_case_ = 21,926; *N*_control_ = 342,747) and a PAD sample of 365,224 individuals (*N*_case_ = 5673; *N*_control_ = 359,551).

Next, we randomly divided these individuals (including cases and controls) into two subsets, comprising 80% and 20% of the sample, respectively. The former (i.e., 80% of the sample) was used as the GWAS discovery sample whereas the latter (i.e., 20% of the sample) was used in the subsequent PRS analysis (see below). GWAS was carried out using BOLT-LMM [20], which is a Bayesian-based linear mixed model (LMM) that allows for the relatedness of individuals by adjusting the population structure and cryptic relatedness. BOLT-LMM was corrected for age, sex, genotype batch, and assessment center and was fitted by random effects of the restricted SNPs (with LD *r*^2^ < 0.9). The SNPs used in BOLT-LMM were calibrated by the LD scores calculated from the 1000 Genomes Project Europeans reference [21]. Genotyped SNPs were imputed according to the Haplotype Reference Consortium reference panel [22]. We further excluded SNPs with minor allele frequency (MAF) < 0.01, imputation INFO score < 0.3, *p*-value of Hardy-Weinberg test < 1 × 10^−6^, minor allele count < 5, or the call rate < 0.05, yielding a final set of 8.54M SNPs. SNP effects (i.e., *β*_BOLT − LMM_) and their associated standard errors (i.e., *SE*_BOLT − LMM_) from BOLT-LMM were converted to a quantitative scale using the approximate approach ${\beta}_{\mathrm{BOLT}-\mathrm{LMM}}^{\prime}\left(\mathrm{or}\ {SE}_{\mathrm{BOLT}-\mathrm{LMM}}^{\prime}\right)=\frac{\beta_{\mathrm{BOLT}-\mathrm{LMM}}\ \left(\mathrm{or}\ {SE}_{\mathrm{BOLT}-\mathrm{LMM}}\right)}{\mu \left(1-\mu \right)}$, where *μ* is the proportion of cases. The remaining individuals (i.e., 20% of cases and 20% of controls) were employed for the PRS analysis (*N*_T2D case_ = 3655, *N*_T2D control_ = 59,801; *N*_PAD case_ = 863, *N*_PAD control_ = 62,593), after excluding the individuals who were genetically related (with a cryptic relatedness *r*^2^ > 0.05) to the individuals of the discovery sample for GWAS.

### Data sources for East Asians

The GWAS summary statistics of T2D [23] and PAD [24] for East Asians were accessed via the BBJ cohort [25]. The GWAS of T2D was a fixed-effect inverse variance-weighted meta-analysis (via METAL [26]) of four Japanese ancestry-based cohorts comprising 36,614 cases and 155,150 controls. The GWAS of PAD included 3593 cases and 208,860 controls and was generated using a LMM via SAIGE [27], adjusted by age, sex, and the top five genetic principal components. Additionally, unrelated East Asian individuals (*N*_T2D case_ = 241, *N*_T2D control_ = 2,483; *N*_PAD case_ = 26, *N*_PAD control_ = 2698) were extracted from UKB (independent from BBJ) for the downstream PRS analysis.

### eQTL summary data

Expression quantitative trait loci (eQTL) are defined as loci which are associated with genetic variants that alter gene expression levels [28]. In our study, we obtained the publicly available blood-based *cis*-eQTL summary data from the eQTLGen Consortium for SMR analysis (see below) [29]. The *cis*-eQTL summary data were generated from 31,684 individuals of European ancestry using a weighted *Z*-score [30] based on a meta-analysis of 37 cohorts comprising 19,250 probes [31].

### Linkage disequilibrium score regression

We performed single-trait and cross-trait LDSC to estimate the liability-scale heritability (*h*^2^) of T2D and PAD as well as their genetic correlation (*r*_*g*_) [32, 33], respectively, according to the population and sample prevalence values of T2D (or PAD) to be 10.00% (or 5.30%) and 6.01% (or 1.55%) in Europeans [34, 35]. The corresponding population and sample prevalence values of T2D (or PAD) were 7.50% (or 4.30%) and 19.09% (or 1.69%) in East Asians (as a test of replicability of the analysis in Europeans) [23, 36]. SNPs were excluded from LDSC if they resided within the major histocompatibility complex (MHC) region (chromosome 6: 28,477,797–33,448,354), were strand-ambiguous (i.e., the A/T and G/C SNPs), or had an MAF less than 0.01. The default 1000 Genomes Project European- and East Asian-based LD score reference panels were employed throughout the analyses. LDSC were performed with and without intercept constraint to explore the impact of potential inflation of the GWAS summary statistics due to the presence of population stratification. The significant genetic correlation was assumed on the basis of a *p*-value < 0.05.

### Mendelian randomization analysis

We applied multiple MR models to investigate the putative causal relationship between T2D and PAD. MR evaluates the causal effect of a risk factor (i.e., exposure) on a target trait (i.e., outcome) using genetic variants as instruments, assuming that the genetic variants concerned are significantly associated with the exposure and have causal effects on the outcome only through the exposure. This latter assumption may be violated due to the presence of horizontal pleiotropy, which occurs if the genetic variants affect the outcome through non-causal pathways [32]. Horizontal pleiotropy can itself be subclassified into *correlated pleiotropy* which is defined as the genetic variants acting on exposure and outcome via shared factors and *uncorrelated pleiotropy* that occurs if the genetic variants act on exposure and outcome via other independent pathways. To distinguish true causality from horizontal pleiotropy, we applied multiple MR and MR-equivalent models employing different assumptions on horizontal pleiotropy, namely inverse variance-weighted (IVW), MR-Egger, weighted mode, weighed median, generalized summary data-based Mendelian randomization (GSMR), the causal analysis using summary effect estimates (CAUSE), and MR-equivalent latent causal variable (LCV) analysis. IVW estimates the Wald ratio for each SNP and calculates the causal estimate using a weighted linear regression which does not correct for horizontal pleiotropy [37]. MR-Egger adds an extra intercept to IVW to weigh the possible deviations attributable to uncorrelated pleiotropy [38]. Weighted mode greatly loosens the assumptions made on correlated and uncorrelated pleiotropy and measures the causal effect only from the most frequent SNP set with consistent effect [39]. The weighted median calculates the causal effect using the weighted median of the SNP ratio under the assumption that most instrumental variants are valid (i.e., more than half the instrumental variants are valid instrumental SNPs) [40]. GSMR is an extension of IVW which applies the heterogeneity in dependent instruments (HEIDI) test to exclude instrumental SNPs with potentially uncorrelated pleiotropic effects [29, 41]. Bayesian-based CAUSE corrects both correlated and uncorrelated pleiotropy via a multivariate linear model adjusted by a joint distribution of instrumental SNPs, assuming that true causality can be attributed to all instrumental SNPs whereas correlated and uncorrelated pleiotropy only affects partial instrumental SNPs [42]. CAUSE further examines the model fitness using the expected log pointwise posterior density (ELPD) by comparing the causal model (i.e., exposure affects outcome via both causal effect and pleiotropic effects), the shared model (i.e., exposure affects outcome only via pleiotropic effects), and the null model (i.e., no causal effect or pleiotropic effects between exposure and outcome). Finally, LCV supposes that the *r*_*g*_ between the two disease entities is regulated by a latent variable with causal effects on both exposure and outcome and evaluates this latent causal variable using the genetic causality proportion (GCP) [43].

MR analyses were carried out using R packages *cause* (version 1.0.0), *LCV*, *gsmr* (version 1.0.9), and *TwoSampleMR* (version 0.5.5). LCV recruits all HapMap3 variants as instrumental SNPs [44]; CAUSE selects independent instrumental SNPs with an arbitrary *p*-value of exposure GWAS < 1 × 10^−5^ by LD pruning; other MR approaches determine independent instrumental SNPs (with GWAS *p*-value < 5 × 10^−8^ or < 1 × 10^−5^ when employing T2D or PAD as exposure) by LD clumping (LD *r*^2^ < 0.05 within 1000-kb windows) using PLINK (v1.90) [45]. Instrumental SNPs were further filtered out if they were located within the MHC region [33], had a MAF less than 0.01, or were nominally significantly associated with the outcome (as potential pleiotropic SNPs).

The putative causal relationship was determined if consistent results were identified by multiple MR models with Bonferroni-corrected *p*-value < 3.85 × 10^−3^ (= 0.05/13, six methods with bi-directional analyses and LCV model). Post hoc power calculations were performed using an online web tool (https://sb452.shinyapps.io/power/) [46]. The causal effects (i.e., *β*) were converted from logit scale to liability scale using the method proposed by Byrne et al. [47]: ${\beta_{\mathrm{x}\mathrm{y}}}_{\mathrm{liability}}=\frac{Z_{K_{\mathrm{x}}}{K}_{\mathrm{y}}\left(1-{K}_y\right)}{Z_{K_{\mathrm{y}}}{K}_{\mathrm{x}}\left(1-{K}_{\mathrm{x}}\right)}{\beta_{\mathrm{x}\mathrm{y}}}_{\mathrm{logit}}$, where *K*_*x*_ and *K*_*y*_ are the population prevalence of exposure and outcome, and ${Z}_{K_x}$ and ${Z}_{K_y}$are the values (heights) of the standard normal distribution at the two population prevalence, respectively. The liability-scale *β* was then transformed to odds ratios (OR), assuming the population prevalence for T2D and PAD to be 10.00% and 5.30% in Europeans [34, 35] and 7.50% and 4.30% in East Asians (as a replicated analysis) [23, 36], respectively.

### Multivariable MR analysis

Multivariable MR (MVMR) is an extension of standard MR that re-estimates the causal effect of exposure on the outcome after adjusting the potential pleiotropic effects from the outcome-related risk factors (i.e., risk factors that may affect the outcome through exposure) [48]. Here, we performed MVMR by considering T2D (or PAD) as the initial exposure, together with additional exposures including total cholesterol (TC) [49, 50], body mass index (BMI) [51, 52], systolic blood pressure (SBP) [53, 54], and smoking initiation [55, 56], which had publicly available GWAS in both Europeans and East Asians. MVMR-based independent instrumental SNPs were selected if they were significantly (*p*-value < 5 × 10^−8^ or < 1 × 10^−5^ when employing T2D or PAD as exposure) associated with at least one exposure. We applied MVMR to ascertain the causality of T2D on PAD (or PAD on T2D) for Europeans and East Asians (for replication), adjusted for each outcome-related risk factor individually as well as for all four risk factors (TC, BMI, SBP, and smoking initiation) acting combinatorically. MVMR was performed using the R package *TwoSampleMR* (version 0.5.5).

### Polygenic risk score analysis

Whenever a genetic correlation between T2D and PAD was identified, PRS analysis was performed to explore whether the aggregated T2D-related genetic effects could predict the status of PAD (and vice versa) based on the genetic profile, using independent European- or East Asian-based samples. SNP sets for PRS calculation were selected by employing 9 different *p*-value cutoffs (i.e., 5 × 10^−8^, 1 × 10^−5^, 1 × 10^−3^, 0.01, 0.05, 0.1, 0.3, 0.5, and 1), which were then filtered by LD clumping (LD *r*^2^ < 0.05 within 1000-kb windows) using PLINK (v1.90) [45]. These independent SNPs were used to calculate PRS through the PLINK “score” function. PRS analysis was performed via logistic regression for the binary T2D status on the scaled PRS for PAD, and the binary PAD status on the scaled PRS for T2D, respectively. The regression model was constructed based on independent samples to test whether PRS from combined effects of multiple T2D risk-associated SNPs can predict the status of PAD, and vice versa. All the regressions were adjusted by age, sex, genotype batch, and assessment center. The variance of the susceptibility to a target trait explained by PRS was quantified by Nagelkerke’s pseudo-coefficient of determination (*R*^2^) [57]. We converted the observed Nagelkerke’s pseudo-*R*^2^ (and their upper and lower bounds of 95% confidence intervals [CI]) to the liability scale, assuming the population and sample prevalence of T2D (or PAD) to be 10.00% (or 5.30%) and 5.76% (or 1.36%) in Europeans [34, 35] and T2D (or PAD) at 7.50% (or 4.30%) and 8.85% (or 0.95%) in East Asians [23, 36], respectively. The standard error of *R*^2^ was estimated using Olkin and Finn’s approximation (${R}_{\mathrm{SE}}^2=\sqrt{\frac{4{R}^2{\left(1-{R}^2\right)}^2{\left(N-K-1\right)}^2}{\left({N}^2-1\right)\left(3+N\right)}}$, where *N* is the sample size and *K* is the number of predictors used in the full model) [58]. The power of each PRS regression was calculated via the R package *AVENGEME* [59].

### Multi-trait analysis of T2D and PAD

To detect risk SNPs shared between T2D and PAD, we performed an inverse variance-weighted cross-trait meta-analysis using MTAG [60], which has an advantage over other meta-analysis tools because MTAG can account for potential sample overlap between GWAS summary data via cross-trait LDSC results. We also calculated “maxFDR” (the approximate upper limit for the false discovery rate [FDR] of the MTAG results) to assess the validity of the assumption that more than 10% of SNPs were causal for each trait. The lower maxFDR value suggests that the MTAG results were unlikely to be false-positive. Novel risk SNPs were identified if they were (1) independent of each other (i.e., LD clumping *r*^2^ < 0.05 in a window of 1000 kb), (2) genome-wide significant (*p*-value < 5 × 10^−8^) associations with the cross-trait shared architecture of T2D and PAD but not single-trait T2D or PAD, and (3) independent from genome-wide significant SNPs of single-trait T2D or PAD (i.e., *r*^2^ < 0.05 in a window of 1000 kb).

### Gene-based association analysis

Whenever a significant shared genetic architecture between T2D and PAD was determined, we then extended the association results from the SNP level to the gene level and applied powerful MAGMA to identify the genes associated with T2D and PAD [61], as well as the cross-trait shared architecture of T2D and PAD in Europeans and East Asians (for replication). A total of 19,259 protein-coding genes (based on NCBI 37.3 build; excluding the MHC genes) were analyzed. SNPs were mapped to a gene if they were located within 50 kb upstream or downstream of the gene length boundary. The 1000 Genomes Project European- and East Asian-based LD reference panels were utilized to correct LD structure. Candidate pleiotropic genes underlying T2D and PAD were identified as those exhibiting associations with the cross-trait shared architecture of T2D and PAD at the FDR 5% significance level.

### Summary data-based Mendelian randomization analysis

When genes were indicated as being significantly associated with the cross-trait shared architecture of T2D and PAD by MAGMA analysis, we further evaluated whether the expression levels of these genes were significantly associated with the cross-trait shared architecture of T2D and PAD using SMR [29]. SMR recruits functional genes as exposure and a focal trait as an outcome, using the top SNPs of eQTLs (*p*-value < 5 × 10^−8^) as instrumental variables, which expects to yield sufficient power to identify significant gene-trait associations [62]. The blood-based *cis*-eQTL summary data were obtained from the eQTLGen Consortium [31]. The HEIDI test [29] was utilized to exclude significant SMR associations due to linkage (i.e., different causal SNPs in LD that influence gene expression and disease separately), rather than causality (i.e., instrumental SNP influences disease via gene expression) or pleiotropy (i.e., instrumental SNP influences both gene expression and disease via shared effects). Significant SMR associations were determined with a Bonferroni-corrected SMR *p*-value (= 0.05/number of tested genes) and a HEIDI *p*-value > 0.01 from a minimum of 10 SNPs. Novel functional genes were ascertained if they exhibited significant associations with the cross-trait shared architecture of T2D and PAD using both MAGMA and SMR.

## Results

### Shared genetics and putative causal relationship between T2D and PAD in Europeans

#### Genetic correlation between T2D and PAD

Using the single-trait LDSC (without constrained intercept, Table 1), we estimated the European-based liability-scale *h*^2^ for T2D and PAD to be 23.47% (*p*-value = 9.42 × 10^−52^) and 10.93% (*p*-value = 8.54 × 10^−9^), respectively. The *h*^2^ values estimated with constrained intercept (T2D: *h*^2^ = 28.08%, *p*-value = 2.29 × 10^−85^; PAD: *h*^2^ = 12.23%, *p*-value = 3.11 × 10^−17^) were compatible with those without constrained intercept, implying mild inflations in these GWAS summary data. Using the cross-trait LDSC with constrained intercept (Table 1), we estimated the *r*_*g*_ between T2D and PAD to be 0.44 (*p*-value = 5.23 × 10^−17^) whereas when we used the cross-trait LDSC without constrained intercept, the *r*_*g*_ between T2D and PAD was estimated to be 0.51 (*p*-value = 9.34 × 10^−15^). These results were held to indicate strong shared genetics between the two conditions in Europeans.

**Table 1:** Genetic correlation between T2D and PAD in Europeans and East Asians

			Constrained intercept		Unconstrained intercept	
			T2D	PAD	T2D	PAD
EUR	Single-trait LDSC	*h*^2^ ± SE	28.08% ± 1.35%	12.23% ± 1.40%	23.47% ± 1.47%	10.93% ± 1.86%
		*P*_h2_	2.29 × 10^−85^	3.11 × 10^−17^	9.42 × 10^−52^	8.54 × 10^−9^
	Cross-trait LDSC	*r*_*g*_ ± SE	0.44 ± 0.05		0.51 ± 0.07	
		*P*_*rg*_	5.23 × 10^−17^		9.34 × 10^−15^	
EAS	Single-trait LDSC	*h*^2^ ± SE	21.48% ± 1.17%	13.58% ± 2.04%	15.12% ± 1.37%	18.83% ± 2.93%
		*P*_h2_	2.45 × 10^−67^	8.34 × 10^−11^	3.61 × 10^−26^	3.44 × 10^−10^
	Cross-trait LDSC	*r*_*g*_ ± SE	0.45 ± 0.07		0.46 ± 0.06	
		*P*_*rg*_	1.16 × 10^−10^		1.67 × 10^−12^	

*PAD* peripheral artery disease, *T2D* type 2 diabetes, *h*^2^ heritability, *r*_*g*_ genetic correlation, *SE* standard error, *P*_*h2*_*p*-value for estimated *h*^2^, *P*_*rg*_*p*-value for estimated *r*_*g*_, *LDSC* linkage disequilibrium score regression, *EUR* Europeans, *EAS* East Asians

#### Putative causal effect of T2D on PAD

**Fig. 1:**
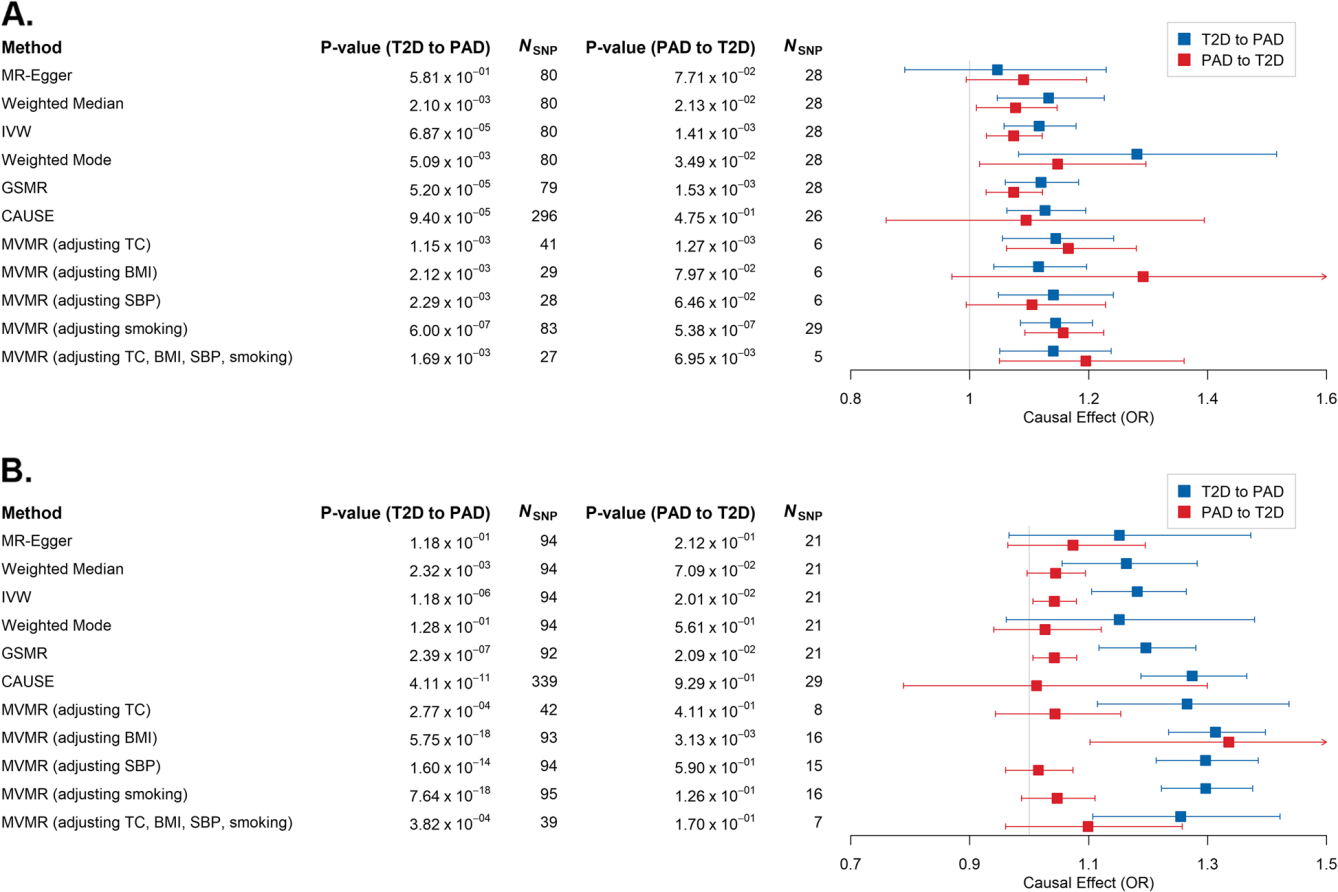
The causal effect (in liability-scale odds ratio [OR]) of type 2 diabetes (T2D) on peripheral artery disease (PAD) as estimated by six Mendelian randomization (MR) models (MR-Egger, weighted median, IVW, weighted mode, GSMR, and CAUSE) in A Europeans and B East Asians. MVMR analyses were performed by adjusting TC [“MVMR (adjusting TC)”], BMI [“MVMR (adjusting BMI)”], SBP [“MVMR (adjusting SBP)”], and smoking [“MVMR (adjusting smoking)”] and by adjusting all these factors jointly [“MVMR (adjusting TC, BMI, SBP, smoking)”] in Europeans and East Asians. Error bars represent the 95% confidence intervals of the estimates. IVW, inverse variance weighted; GSMR, generalized summary data-based Mendelian randomization; CAUSE, causal analysis using summary effect estimates; MVMR, multivariable MR; TC, total cholesterol; BMI, body mass index; SBP, systolic blood pressure

The application of multiple MR methods provided suggestive evidence for a causal effect of T2D on PAD (Fig. 1A, Additional file 1: Table S2 and S3) in Europeans. We observed that four out of seven MR models (with the exception of MR-Egger [*p*-value = 0.58], weighted mode [*p*-value = 5.09 × 10^−3^], and LCV [*p*-value = 0.28]) surpassed the Bonferroni-corrected level of significance (*p*-value < 3.85 × 10^−3^), with estimated liability-scale odds ratios (ORs) at ~ 1.14 on average, suggesting that T2D patients had approximately 1.14 times the risk (mean standard error = 5.83 × 10^−2^) of developing PAD compared to healthy individuals. The power of MR models was evaluated to be in the range of 78–100% (except for the MR-Egger model that failed to reach the 5% level of significance), corresponding to the causal effect estimated between 1.12 and 1.28 (Additional file 2: Fig. S1). These results suggested the sufficiency of study power of our GWAS summary statistics to identify causality between T2D and PAD, although the power of MR models for PAD on T2D is relatively limited compared to those for T2D on PAD. Horizontal pleiotropy would appear to have had a limited impact on this putative causal relationship, since MR-Egger suggested a close-to-zero intercept (intercept = 6.75 × 10^−3^, *p*-value = 0.41) while CAUSE indicated a better fit for the causal model compared to either the sharing model (ELPD *p*-value = 4.77 × 10^−3^) or the null model (ELPD *p*-value = 8.57 × 10^−3^). Furthermore, recruiting weaker instrumental SNPs (*p*-value < 1 × 10^−5^) for the CAUSE model is unlikely to have affected the accuracy of the MR estimates, as similar results (Additional file 1: Table S2 and S3) were obtained in a parallel CAUSE analysis utilizing a stricter *p*-value threshold of 5 × 10^−8^. Applications of MVMR analyses found that the putative causal effect of T2D on PAD was largely unchanged after adjusting for TC, BMI, SBP, and smoking individually, as well as adjusting for these risk factors as a whole (Fig. 1A and Additional file 1: Table S4). Furthermore, there was no consistent causal effect of PAD on T2D using MVMR after adjusting for the same risk factors (Fig. 1A and Additional file 1: Table S4).

#### Higher PRS for T2D in PAD patients than in non-PAD controls

**Fig. 2:**
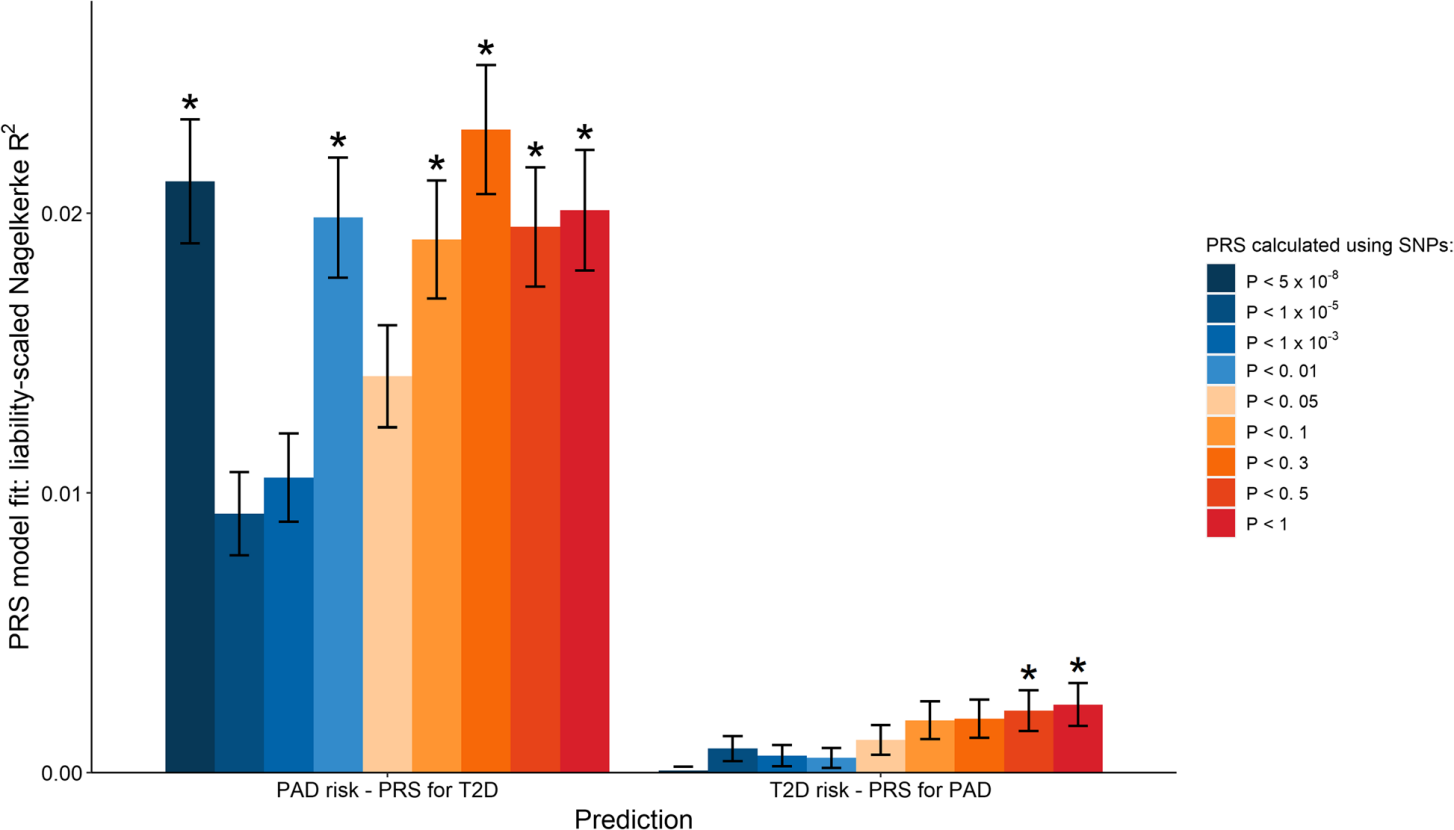
European-based liability-scaled Nagelkerke’s *R*^2^ presents the goodness of fit of the logistic regression model to peripheral artery disease (PAD) status with polygenic risk score (PRS) for type 2 diabetes (T2D) (“PAD status – PRS for T2D”) and the goodness of fit of the logistic regression model to T2D status with PRS for PAD (“T2D status – PRS for PAD”). Error bars represent the 95% confidence intervals of the estimated Nagelkerke’s *R*^2^. Asterisk represents a significant coefficient (*p*-value < 2.78 × 10^−3^ = 0.05/18, Bonferroni correction) of the PRS terms in regression

Irrespective of the *p*-value cutoffs in the PRS calculations, we observed consistently higher PRS for T2D in patients with PAD compared to those without PAD (Additional file 1: Table S5). The increased *p*-value cutoffs appeared to drive up the values of the liability-scaled Nagelkerke’s pseudo-*R*^2^ (except for the results below *p*-value < 5 × 10^−8^), suggesting that PRS for T2D calculated using additional SNPs could explain more of the variance of PAD, consistent with the expectation that PRS regression (Additional file 2: Fig. S2) had better predictive power when additional SNPs were included and when the sample size of GWAS summary statistics was increased. This notwithstanding, the reverse (PRS for PAD in the prediction of T2D status) was non-significant (Additional file 1: Table S6). Liability-scaled Nagelkerke’s pseudo-*R*^2^ of PRS for PAD on T2D status were also significantly lower (*p*-value_paired *t*-test_ = 6.15 × 10^−6^) than those estimations of PRS for T2D on PAD status (Fig. 2). These results provided further evidence in support of the genetic overlap between T2D on PAD in Europeans.

#### Novel SNPs and candidate genes associated with the shared genetics of T2D and PAD

**Fig. 3:**
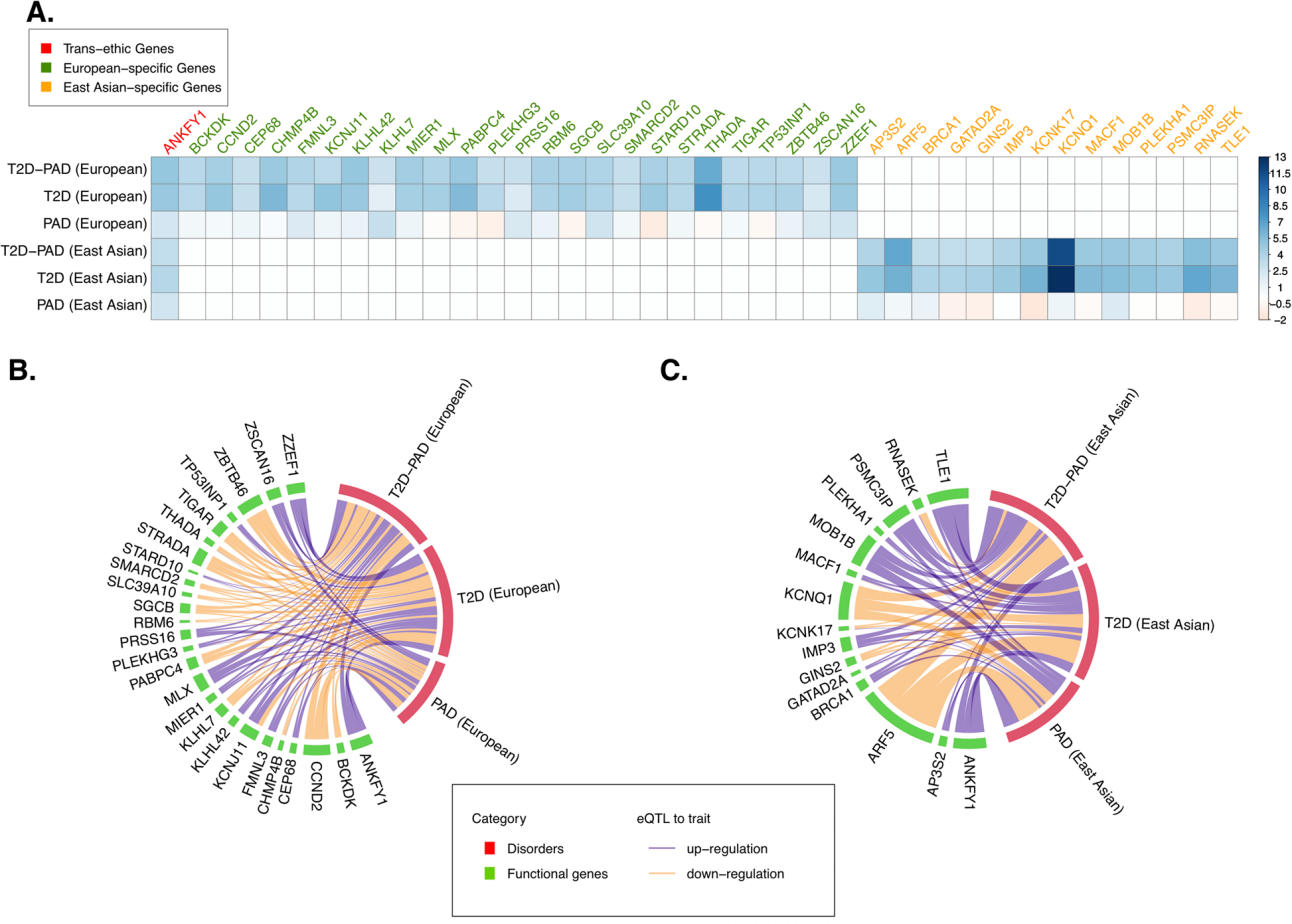
Genes significantly associated with type 2 diabetes (T2D), peripheral artery disease (PAD), or cross-trait shared architecture of T2D and PAD identified by MAGMA or SMR analysis in Europeans and East Asians. **A** The genes significantly associated with T2D, PAD, or cross-trait shared architecture of T2D and PAD identified by multi-marker analysis of genomic annotation (MAGMA) in Europeans and East Asians, and the genes associated with T2D, PAD, or cross-trait shared architecture of T2D and PAD identified by summary data-based Mendelian randomization (SMR) in **B** Europeans and **C** East Asians. The color bar in A represents the *z*-score in MAGMA analysis. The width of the line in **B** and **C** denotes the strength of the SMR association. In **B** and **C**, lines in purple and orange represent respectively up- and downregulated genes associated with the increasing risk of the disease in question

Performing a meta-analysis of the T2D and PAD GWAS data using MTAG revealed two novel SNPs, rs927742 and rs1734409 (Additional file 1: Table S7) that were significantly (*p*-value < 5 × 10^−8^) associated with the cross-trait shared architecture of T2D and PAD (MTAG MaxFDR = 4.54 × 10^−7^) and independent from the top SNPs of single-trait T2D or PAD. We also applied gene-based MAGMA analysis to identify a total of 898 FDR significant genes associated with the cross-trait shared architecture of T2D and PAD (Additional file 1: Table S8 and Fig. 3A). Among them, 136 genes were only associated with the cross-trait shared architecture of T2D and PAD (i.e., novel pleiotropic genes); the rest of the genes were additionally associated with T2D (*N* = 761; i.e., T2D-specific genes) or both T2D and PAD (*N* = 1; i.e., pleiotropic gene). Furthermore, SMR and HEIDI analysis implied 26 genes (out of 898 MAGMA genes; three as novel pleiotropic genes and 23 as T2D-specific genes) whose expression levels were significantly (*p*-value < 8.05 × 10^−5^ with Bonferroni correction) associated with the risk of the cross-trait shared architecture of T2D and PAD due to either pleiotropy or causality (Additional file 1: Table S9 and Fig. 3B).

### Replicability of the putative causal relationship between T2D and PAD in East Asians

In East Asians, the *h*^2^ values for T2D and PAD were estimated to be 15.12% (*p*-value = 3.61 × 10^−26^) and 18.83% (*p*-value = 3.44 × 10^−10^) without constrained intercept and 21.48% (*p*-value = 2.45 × 10^−67^) and 13.58% (*p*-value = 8.34 × 10^−11^) with constrained intercept (Table 1). The *r*_*g*_ values between T2D and PAD were estimated to be 0.45 (*p*-value = 1.16 × 10^–10^) and 0.46 (*p*-value = 1.67 × 10^−12^) with and without constrained intercept, respectively. These results were largely similar to those derived from Europeans, indicating strong shared genetics between T2D and PAD in different ethnicities.

We also identified a putative causal effect of T2D on PAD in East Asians (Fig. 1B, Additional file 1: Table S10 and S11), with five of seven MR models surpassing the Bonferroni-corrected threshold (except for MR-Egger [*p*-value = 0.12] and weighted mode [*p*-value = 0.13]). The estimated ORs for the causality of T2D on PAD in East Asians ranged from 1.15 to 1.27, which were slightly higher than those noted in Europeans. The power of MR models in East Asians was estimated to be in excess of 99% (Additional file 2: Fig. S1) for T2D on PAD, slightly higher than those in Europeans, while the power of MR models in East Asians for PAD on T2D was still significantly lower than those for T2D on PAD. These results were deemed to be less likely to be affected by horizontal pleiotropy (MR-Egger intercept = 2.54 × 10^−3^, *p*-value = 0.76; CAUSE causal model vs. sharing model ELPD *p*-value = 2.63 × 10^−4^ and vs. null model ELPD *p*-value = 1.60 × 10^−5^) or the application of weaker instrumental SNPs (similar results from the parallel CAUSE model using instrumental SNPs with *p*-value < 5 × 10^−8^; Additional file 1: Table S10). Additionally, the causal effect of T2D on PAD was less likely to be influenced by TC, BMI, SBP, or smoking, according to the MVMR results (Fig. 1B and Additional file 1: Table S12).

We next attempted to investigate whether the aggregated T2D (or PAD)-related genetic effects could be used to predict the status of PAD (or T2D) using PRS regression (Additional file 1: Table S13 and S14), but failed to identify significant results, probably on account of the insufficient sample size limiting the statistical power of regression (Additional file 2: Fig. S2). Nevertheless, we still observed significantly higher (*p*-value_paired *t*-test_ = 1.98 × 10^−3^) liability-scaled Nagelkerke’s pseudo-*R*^2^ values estimated from the regression of PRS for T2D on PAD status than those of PRS for PAD on T2D status, suggesting that PAD maybe genetically a consequence of T2D but not the other way around.

We performed MTAG analysis but did not identify any novel genetic loci significantly associated with the cross-trait shared architecture of T2D and PAD in East Asians (MTAG MaxFDR = 4.54 × 10^−7^). Whereas gene-based MAGMA analysis found 372 genes exhibiting significant associations (FDR < 0.05) with cross-trait shared architecture of T2D and PAD (all T2D-specific genes; see Additional file 1: Table S15 and Fig. 3A), 15 of which were further observed as significant associations by SMR and HEIDI analysis (Bonferroni-corrected SMR *p*-value < 1.92 × 10^−4^; Additional file 1: Table S16 and Fig. 3C).

### Common and distinct mechanisms underlying the co-occurrence of T2D and PAD between Europeans and East Asians

**Fig. 4:**
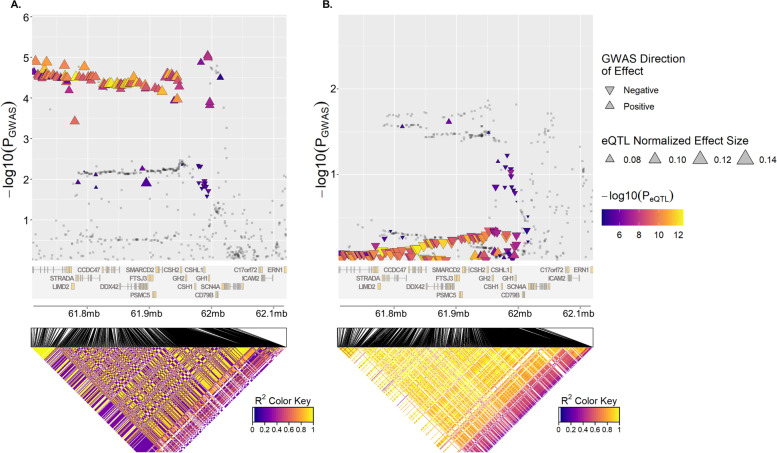
The genomic region near to *SMARCD2* gene was used as an example to illustrate the different genetic structures between Europeans (**A**) and East Asians (**B**). *SMARCD2* was found to be significantly associated with the cross-trait shared architecture of type 2 diabetes (T2D) and peripheral artery disease (PAD) in Europeans but not in East Asians by MGAMA and SMR analysis. The dot and the triangle represent respectively *p*-values and effect sizes of SNPs in GWAS and eQTL. The directions of the triangles indicate the direction of SNPs in GWAS. Most of the SNPs plotted in **A** and **B** show opposite GWAS directions. The heatmap represents the linkage disequilibrium (LD) of the SNPs, which are obviously stronger in East Asians than in Europeans. GWAS, genome-wide association studies; eQTL, expression quantitative trait loci

Gene-based analyses (i.e., MAGAMA and SMR) in Europeans and East Asians consistently identified the *ANKFY1* gene as being significantly associated with the cross-trait shared architecture of T2D and PAD in both Europeans (*p*-value_MAGAMA_ = 3.28 × 10^−7^; *β*_SMR_ = 0.44, *p*-value_SMR_ = 1.18 × 10^−5^) and East Asians (*p*-value_MAGAMA_ = 6.90 × 10^−4^; *β*_SMR_ = 0.42, *p*-value_SMR_ = 1.83 × 10^−4^). MAGAMA and SMR additionally identified 25 and 14 genes which were significantly associated with cross-trait shared architecture of T2D and PAD in Europeans (FDR_MAGAMA_ < 2.49 × 10^−3^; *p*-value_SMR_ < 8.05 × 10^−5^) and East Asians (FDR_MAGAMA_ < 1.06 × 10^−3^; *p*-value_SMR_ < 1.94 × 10^−4^), respectively. These genes showed a significant (*p*-value = 1.53 × 10^−2^) enrichment in their protein-protein interactions (PPI) with each other according to STRING (Additional file 2: Fig. S3) [63], including seven interactions between genes significantly associated with the cross-trait shared architecture of T2D and PAD in Europeans and genes associated with the cross-trait shared architecture of T2D and PAD in East Asians (e.g., interactions of *STARD10* [European-specific] with *AP3S2* [East Asian-specific], and *KCNJ11* [European-specific] with *KCNQ1* [East Asian-specific]). These findings point to the existence of common trans-ethnic mechanisms underlying T2D and PAD. Nevertheless, we also noted distinct mechanisms underlying the co-occurrence of T2D and PAD between Europeans and East Asians, which might be attributed to the different LD structures in the two ethnicities (Fig. 4 taking gene *SMARCD2* as an example).

## Discussion

In this study, we systematically investigated the shared genetic architecture between T2D and PAD in Europeans and East Asians by leveraging their large-scale GWAS summary statistics. Our study disclosed a putative causal effect of T2D upon PAD in both Europeans and East Asians and identified several novel loci/functional genes that might be relevant to the shared genetics underlying T2D and PAD. We additionally used MVMR analysis to exclude an underlying heritable trait as the basis of the causal relationship between T2D and PAD and indicated that the causal effect of T2D on PAD is not influenced by TC, BMI, SBP, or smoking initiation. All these traits are generally considered to be factors that are associated with a high risk of PAD and T2D, and they are known to be related with dysfunction of lipoproteins or hemostasis [64, 65], etc. Indeed, recent studies have revealed lipoproteins and hemostatic factors to be risk factors for PAD [66–68]. Our study may indirectly indicate that neither the lipoproteins nor hemostatic factors have much influence on the causal relationship between T2D and PAD. Taken together, this study not only improves our understanding of the genetic etiology shared by T2D and PAD but also provides evidence to support the concept of genetically screening T2D patients in order to pre-symptomatically detect/prevent PAD.

To our knowledge, this is the first study to investigate the shared genetic architecture between T2D and PAD, although a significant clinical/phenotypic correlation has been previously reported [69, 70]. One of the main aims of this study was to investigate the shared genetic relationship between T2D and PAD in Europeans and East Asians. On the basis of an online LDSC power calculation (https://nealelab.github.io/UKBB_ldsc/viz_sampsize.html), we believe our input GWAS summary statistics have sufficient power (> 95%) to uncover shared genetic associations between T2D and PAD in either Europeans or East Asians. Here, we observed a genetic correlation between the two disease entities in both Europeans and East Asians. The estimated *h*^2^ of T2D and PAD, as well as the *r*_*g*_ between T2D and PAD in Europeans, were largely similar to those in East Asians, suggesting a strong genetic correlation between T2D and PAD in both ethnicities.

In both Europeans and East Asians, we applied multiple MR methods and consistently identified the putative causal effect of T2D in relation to PAD, suggesting a common causal genetic relationship between T2D and PAD in Europeans and East Asians. Among multiple MR methods, CAUSE and LCV were the only models capable of distinguishing both correlated and uncorrelated pleiotropy from causality. LCV can yield conservative results when the genetic correlation is mild and cannot measure the extent of the causal effect directly. Other MR models can only deal with uncorrelated pleiotropy or partially correlated pleiotropy. Therefore, we consider CAUSE to be a prior MR method compared to other models. In our study, CAUSE showed consistent evidence for a putative causal effect of T2D on PAD, indicating that our findings are credible. Furthermore, MVMR analysis indicated that the putative causal effect of T2D on PAD is less likely to be influenced by TC, BMI, SBP, and smoking which have generally been considered to be common factors associated with the increased risk of PAD [71–76]. We further identified a higher PRS for T2D among PAD patients compared to non-PAD individuals using PRS regression, in line with previous studies that have reported T2D as a risk factor for arterial disease (such as coronary artery disease and arterial stiffness [77, 78]). Taken together, we conclude that T2D is a causal risk factor for PAD, although further studies with larger sample sizes (particularly with reference to additional PAD patients) will be required to establish this causal relationship conclusively. Nevertheless, several MR models (MR-Egger, weighted mode, and LCV) failed to fully distinguish causality from horizontal pleiotropy, after allowing for Bonferroni correction. The underlying reason may be the limitations inherent to these models. For instance, MR-Egger may inflate type I errors when horizontal pleiotropy from instrumental SNPs occurs via the same confounder [79]. The weighted mode is thought to be less powerful in comparison with other MR methods as it uses a smaller set of instrumental SNPs [80]. The standard error of GCP statistics produced by LCV analysis may be enhanced when analyzing the trait pair with mild or moderate genetic correlation [43].

Previous studies have provided support for the conclusion that while East Asians are more prone to T2D than Europeans [1, 81, 82], the prevalence of PAD in T2D patients is lower in East Asians than in Europeans [83]. The underlying reason has remained unclear, although genetic factors are thought to make important contributions to the ethnic disparities in relation to T2D and PAD prevalence [83–86]. Herein, we provide the first evidence for the common and distinct mechanisms underlying the shared genetics of T2D and PAD between Europeans and East Asians. We used MAGMA and SMR analyses to identify the *ANKFY1* gene as showing significant associations with the cross-trait shared architecture of T2D and PAD in both Europeans and East Asians. *ANKFY1* is thought to play a role in angiogenesis on the basis of experiments in endothelial cells [87, 88] and has been reported as a gene conferring T2D risk in a GWAS study [34]. Another study has identified a homozygous missense mutation in *ANKFY1* to be a potential cause of nephrotic syndrome [89], a disease commonly comorbid with diabetes and cardiovascular disease [90, 91]. Moreover, protein-protein interaction analysis provided further evidence for common mechanisms shared between Europeans and East Asians by revealing gene-gene interactions underlying the comorbidity of T2D and PAD, specifically the interactions between *STARD10* (European-specific) and *AP3S2* (East Asian-specific) and between *KCNJ11* (European-specific) and *KCNQ1* (East Asian-specific). In the context of the interaction of *STARD10* (European-specific) and *AP3S2* (East Asian-specific), *STARD10* is a phospholipid transfer protein whose expression in isolated pancreatic islets has been found to influence the production and processing of insulin in the mouse [92]. Prior studies indicated that *STARD10* harbors SNPs associated with T2D [93, 94]. *AP3S2* encodes a protein involved in protein transport whose variants are associated with the risk of T2D in South Asians and Japanese [95, 96]. Other studies have implicated *AP3S2* as being important for the pathogenesis of T2D [97–101]. Regarding the interaction between *KCNJ11* and *KCNQ1*, both genes contribute to potassium channels whereas genetic variants located near both *KCNJ11* and *KCNQ1* are known to be significantly associated with T2D [9, 102]. Here, we revealed their potential roles in the occurrence of PAD in T2D patients in both Europeans and East Asians. In addition, numerous genes identified as being associated with the cross-trait shared architecture of T2D and PAD in Europeans have similar functions to those in East Asians. For example, *TP53INP1* (European-specific) and *MOB1B* (East Asian-specific) both have functions related to cell migration and death [103, 104]; *CHMP4B* (European-specific) and *AP3S2* (East Asian-specific) are involved in protein transport and intracellular trafficking [105, 106]. These genes were reported as harboring SNPs significantly associated with T2D [1, 107, 108]. Here, they are suggestive of common genetic mechanisms of comorbid T2D and PAD between the two populations.

The distinct genetic mechanisms of co-occurrence of T2D and PAD between Europeans and East Asians may be explicable in terms of the discrepant prevalence of T2D/PAD between Europeans and East Asians. The discrepant prevalence of T2D between ethnic groups may be attributed to insulin resistance and β-cell dysfunction [109]. T2D in Europeans tends to be caused by obesity and insulin resistance whereas T2D in East Asians, who are generally characterized by a leaner body mass, is caused by more severe β-cell dysfunction [82]. Our study potentially supports the view that the distinct genetic mechanisms of co-occurrence of T2D and PAD could be due, at least in part, to the differences in the prevalence of insulin resistance and β-cell dysfunction between the two populations. For instance, we identified the insulin resistance-related gene *TIGAR* in Europeans and the β-cell dysfunction-related gene *TLE1* in East Asians as being associated with the cross-trait shared architecture of T2D and PAD (Additional file 1: Table S9) [110, 111]. *TIGAR*, activated by p53, promotes insulin resistance in the rat whereas *TLE1* has been found to play a role in inducing pancreatic β-cells and converting α-cells to β-cells [110, 111]. Further studies are required to explore the different roles that insulin resistance and β-cell dysfunction play in causing T2D-PAD comorbidity in Europeans and East Asians.

Our findings are potentially important for pre-symptomatic screening in order to prevent the occurrence of PAD in those T2D patients with genetic defects associated with PAD. Identifying incipient PAD among T2D patients and carrying out clinical interventions prior to the onset of arterial disease has the potential to dramatically improve the life quality and prognosis of T2D patients [112]. Our results could lead to the early-stage genetic screening for PAD in T2D patients thereby offering more effective diagnostic and therapeutic approaches for both diseases. Additionally, our study revealed the putative shared/distinct mechanisms underlying the causal relationship between T2D and PAD in Europeans and East Asians, which highlights the need for personalized intervention in designing therapies for PAD in T2D patients with different ethnic backgrounds.

### Study limitation

Our study has several limitations. First, the sample size of East Asians in the UKB cohort is insufficient, thereby limiting the power of the study to confirm the putative causal relationship between T2D and PAD in East Asians via PRS analysis. Clearly, the results of this study should be replicated on a much larger group of East Asians. Second, neither European- nor East Asian-based PAD GWAS have enough instrumental SNPs (*p*-value < 5 × 10^−8^) for testing with some MR models. Alternatively, we relaxed the *p*-value threshold and recruited the “proxy” instrumental SNPs (with *p*-value < 1 × 10^−5^), which may have led to a violation of MR assumptions. Nevertheless, this influence is likely to be negligible as we obtained very similar results when applying multiple MR methods for sensitivity analysis. Third, the SMR results for East Asian-based GWAS were probably underpowered, as the eQTL summary data from eQTLGen were of European descent, which hampered our ability to identify trans-ethnic functional genes underlying T2D and PAD. Fourth, we excluded SNPs in the MHC region from all our analyses because of the complicated LD pattern within the MHC region; this could have led to an underestimation of the shared genetic basis between T2D and PAD. Finally, the classification of ICD-10-based T2D cases in our study may be less precise because we cannot filter out some rare cases of maturity-onset diabetes of the young (MODY). Nevertheless, as the proportion of MODY comprises only 1-5% of total diabetes [113], we believe such influence is likely to be minimal.

## Conclusions

In conclusion, our study has provided the first evidence for a trans-ethnic genetic association between T2D and PAD, a trans-ethnic putative causal effect of T2D on PAD, and the identification of multiple genes which represent candidates for involvement in the shared genetic etiology between T2D and PAD in different ethnic groups. Acquiring a better understanding of the shared genetic architecture underlying T2D and PAD should help to improve clinical treatment and disease prevention of PAD in patients with T2D.

Web resources: GWAS summary statistics from Biobank Japan, http://jenger.riken.jp/en/; BOLT-LMM (v2.3.4), https://alkesgroup.broadinstitute.org/BOLT-LMM/; eQTLGen Consortium, https://eqtlgen.org/cis-eqtls.html; 1000 Genomics LD score reference panels, https://alkesgroup.broadinstitute.org/LDSCORE/; STRING, https://string-db.org/; and Online tool for MR power calculation, https://sb452.shinyapps.io/power/.

## Supplementary Information

**Additional file 1: Table S1**. ICD codes of PAD and T2D. **Table S2**. MR results between T2D and PAD in Europeans. **Table S3**. MR results between T2D and PAD in Europeans (without excluding potential pleiotropy SNPs). **Table S4**. MVMR results between T2D and PAD adjusted by potential risk factors in Europeans. **Table S5**. Summary of PRS regression of PRS for T2D on PAD status in Europeans. **Table S6**. Summary of PRS regression of PRS for PAD on T2D status in Europeans. **Table S7**. Novel SNPs underlying the shared genetics of T2D and PAD in Europeans detected by MTAG. **Table S8**. Candidate genes associated with comorbid T2D and PAD in Europeans detected by MAGMA. **Table S9**. Significant SMR associations of gene expression (from Table S8) with T2D, PAD, and their cross-trait. **Table S10**. MR results between T2D and PAD in East Asians. **Table S11**. MR results between T2D and PAD in East Asians (without excluding potential pleiotropy SNPs). **Table S12**. MVMR results between T2D and PAD adjusted by potential risk factors in East Asians. **Table S13**. Summary of PRS regression of PRS for T2D on PAD status in East Asians. **Table S14**. Summary of PRS regression of PRS for PAD on T2D status in East Asians. **Table S15**. Candidate genes associated with comorbid T2D and PAD in East Asians detected by MAGMA. **Table S16**. Significant SMR associations of gene expression (from Table S15) with T2D, PAD, and their cross-trait. (XLS 383 kb)

**Additional file 2: Figure S1**. Estimated powers of European- or East Asian-based MR models for the causality between T2D and PAD. **Figure S2**. The power of PRS regression regarding SNP *p*-value thresholds of different discovery GWAS. **Figure S3**. The protein-protein interaction (PPI) network for candidate genes.

## Data Availability

The datasets analyzed during the current study are available in the BBJ, http://jenger.riken.jp/en/.

## Abbreviations

T2D: Type 2 diabetes
PAD: Peripheral artery disease
PRS: Polygenic risk score
MAGMA: Multi-marker analysis of genomic annotation
SMR: Summary data-based Mendelian randomization
GWAS: Genome-wide association studies
LDSC: Linkage disequilibrium score regression
PCs: Principal components
HM 3: HapMap 3SNP: single nucleotide polymorphism
MR: Mendelian randomization
UKB: UK Biobank
BBJ: BioBank Japan
MVMR: Multivariable Mendelian randomization analysis
MTAG: Multi-trait analysis of GWAS
LMM: Linear mixed model
MAF: minor allele frequency
eQTL: Expression quantitative trait loci
*h*^2^: Heritability
*r*_*g*_: Genetic correlation
MHC: Major histocompatibility complex
IVW: Inverse variance-weighted
GSMR: Generalized summary data-based Mendelian randomization
CAUSE: Causal analysis using summary effect estimates
LCV: Latent causal variable
HEIDI: Heterogeneity in dependent instruments
ELPD: Expected log pointwise posterior density
GCP: Genetic causality proportion
TC: Total cholesterol
BMI: Body mass index
SBP: Systolic blood pressure
CI: Confidence intervals
